# Designing high-quality implementation research: development, application, feasibility and preliminary evaluation of the implementation science research development (ImpRes) tool and guide

**DOI:** 10.1186/s13012-019-0897-z

**Published:** 2019-08-14

**Authors:** Louise Hull, Lucy Goulding, Zarnie Khadjesari, Rachel Davis, Andy Healey, Ioannis Bakolis, Nick Sevdalis

**Affiliations:** 1 Centre for Implementation Science, Health Service and Population Research Department, King’s College London, London, UK; 2 School of Health Sciences, University of East Anglia, Norwich Research Park, Norwich, UK; 3 King’s Health Economics, Institute of Psychiatry, Psychology and Neuroscience, King’s College London, London, UK; 4 Department of Biostatistics and Health Informatics, Institute of Psychiatry, Psychology and Neuroscience, King’s College London, London, UK

**Keywords:** Implementation science, Implementation research, Design guidelines, Methods

## Abstract

**Background:**

Designing implementation research can be a complex and daunting task, especially for applied health researchers who have not received specialist training in implementation science. We developed the Implementation Science Research Development (ImpRes) tool and supplementary guide to address this challenge and provide researchers with a systematic approach to designing implementation research.

**Methods:**

A multi-method and multi-stage approach was employed. An international, multidisciplinary expert panel engaged in an iterative brainstorming and consensus-building process to generate core domains of the ImpRes tool, representing core implementation science principles and concepts that researchers should consider when designing implementation research. Simultaneously, an iterative process of reviewing the literature and expert input informed the development and content of the tool. Once consensus had been reached, specialist expert input was sought on involving and engaging patients/service users; and economic evaluation. ImpRes was then applied to 15 implementation and improvement science projects across the National Institute of Health Research (NIHR) Collaboration for Leadership in Applied Health Research and Care (CLAHRC) South London, a research organisation in London, UK. Researchers who applied the ImpRes tool completed an 11-item questionnaire evaluating its structure, content and usefulness.

**Results:**

Consensus was reached on ten implementation science domains to be considered when designing implementation research. These include implementation theories, frameworks and models, determinants of implementation, implementation strategies, implementation outcomes and unintended consequences. Researchers who used the ImpRes tool found it useful for identifying project areas where implementation science is lacking (median 5/5, IQR 4–5) and for improving the quality of implementation research (median 4/5, IQR 4–5) and agreed that it contained the key components that should be considered when designing implementation research (median 4/5, IQR 4–4). Qualitative feedback from researchers who applied the ImpRes tool indicated that a supplementary guide was needed to facilitate use of the tool.

**Conclusions:**

We have developed a feasible and acceptable tool, and supplementary guide, to facilitate consideration and incorporation of core principles and concepts of implementation science in applied health implementation research. Future research is needed to establish whether application of the tool and guide has an effect on the quality of implementation research.

## Background

Evidence-based and cost-effective interventions consistently fail to be implemented into routine practice and policy [1], and even when such interventions are implemented, this is an effortful, unpredictable and typically slow process [2]. As a result, despite increasing pressure to improve the safety and quality of financially overstretched healthcare services, patients fail to receive optimal care and healthcare organizations fail to benefit from cost saving opportunities [1]. Over the past few years, increased and focused efforts to close the *evidence*-*to*-*practice gap* have resulted in further recognition of the importance of implementation science as a conceptual and methodological approach to translate evidence into routine practice [1, 3, 4].

However, despite the rapid growth of implementation science, designing implementation research remains a complex and daunting task for health researchers who have not completed specialist training in implementation science. Crable et al. [5] operationalized Proctor et al.’s recommended *ten key ingredients* for writing implementation research grant proposals [6] to quantitatively evaluate the quality of proposals on ten criteria. Thirty pilot grant applications submitted to a call for implementation and improvement science projects at an academic medical center were assessed. Crable et al. reported that most proposals assessed performed poorly on most of the ten criteria [5]. For example, 67% of proposals failed to identify and describe the implementation strategies to be used and/or incorrectly described the intervention as an implementation strategy. Furthermore, 70% of proposals failed to describe implementation or improvement science related outcomes and/or failed to link outcomes to the proposed study aims and/or the unit of analysis was inappropriate for the proposed study.

The challenge of designing implementation research is exacerbated by the fact that implementation research cuts across diverse scientific fields, resulting in the inevitable difficulty of identifying, appraising and synthesising relevant literature to inform design decisions. A recently published editorial in *Implementation Science* highlighted the need for capacity building initiatives in the research and practice of implementation to fulfil the demand for expertise in implementation science [7].

Concerns regarding the lack of guidance for designing implementation research have been raised and, to a certain extent, are being addressed. For example, Waltz et al. highlighted that guidance regarding how best to select implementation strategies is lacking [8]. Similarly, lack of guidance on how to select appropriate implementation theories and frameworks has been highlighted and efforts to develop such guidance is currently underway [9].

Whilst these efforts are worthwhile and necessary to advance the science of implementation, a tool consolidating design guidance, to the best of our knowledge, does not currently exist. As a result, healthcare researchers without access to specialist implementation science expertise are tasked with identifying and assimilating design guidance and recommendations reported across a wide range of journals when designing implementation research, or research with substantial implementation components. This is a challenging task, not always successfully accomplished—as evidenced by the aforementioned literature.

To address this challenge, we report the development, application, feasibility and preliminary evaluation of the Implementation Science Research Development (ImpRes) tool and supplementary guide to provide health researchers with a step-by-step approach to designing high-quality implementation research. Specifically, we aimed to (1) identify the core principles and concepts that research teams should consider when designing high-quality implementation research and (2) identify and synthesize key methodological/conceptual literature containing guidance and recommendations for designing and evaluating implementation research. Based on the above aims, we aimed to develop, apply and evaluate a tool that guides researchers through the key principles and research design considerations of implementation science when designing studies. The ImpRes tool and guide aim to enable research teams to design high-quality implementation research and as a result more effectively implement evidence-based interventions into routine practice, thereby reducing research waste, and improving health outcomes.

## Methods

**Fig. 1:**
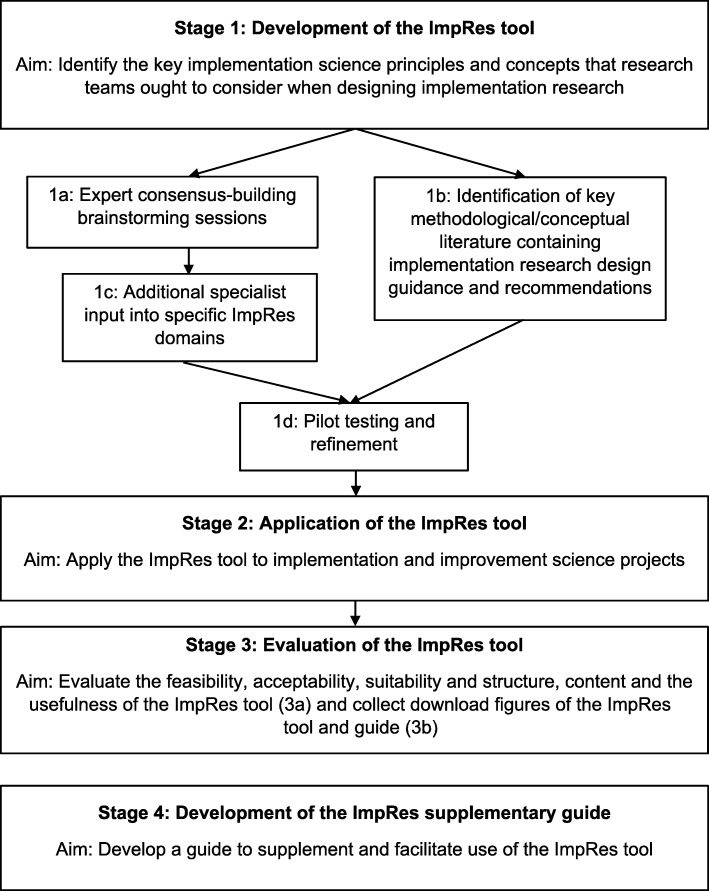
Development of the ImpRes tool and supplementary guide

A multi-stage, multi-method approach was used to develop and evaluate the ImpRes tool and the subsequent development of its supplementary guide (see Fig. 1).

### Stage 1: development of the ImpRes tool (July 2015–May 2016)

Development of the ImpRes tool began in July 2015. The starting point and primary factor motivating the development of the tool was firstly to evaluate the degree to which the core principles and concepts of implementation science were embedded into research projects conducted within a research organisation—the National Institute for Health Research (NIHR) Collaboration for Leadership in Applied Health Research and Care (CLAHRC) South London; and secondly to provide implementation research support to research teams as required. After an initial review of the literature and consultation with experts in the field, it was evident that a tool (or framework) did not exist to allow for this form of evaluation. The NIHR CLAHRC South London is a collaborative partnership between two universities (King’s College London and St George’s, University of London), four geographically surrounding health service organisations (Guy’s and St Thomas’ NHS Foundation Trust, King’s College Hospital NHS Foundation Trust, St George’s University Hospitals NHS Foundation Trust and South London and Maudsley NHS Foundation Trust) and two other collaborating organisations (NHS England-funded Health Innovation Network (HIN), south London’s academic health science network; and King’s Health Partners, the academic health sciences centre in south-east London). NIHR CLAHRC South London was established in 2014 as one of 13 such research organisations supporting the conduct and application of applied health research across England; it has a catchment area of approximately 3.5 million people living and/or using the health services within south London, England. NIHR CLAHRC South London conducts research spanning a diversity of healthcare areas including clinical (e.g. maternity and women’s health and psychosis) and public health. In addition to our collaborators within the London geography, a significant body of the research conducted by NIHR CLAHRC South London researchers is conducted with national and international collaborators. For example, researchers in our ‘diabetes theme’ are leading a hybrid effectiveness-implementation study runs across the UK and US treatment sites [10]. Similarly, a wide-ranging portfolio of hybrid effectiveness-implementation and implementation research is being conducted across the research infrastructure, in many cases with international collaborators [11], as well as methodological implementation science research [12]. The NIHR CLAHRC South London includes the Centre for Implementation Science (CIS), which consists of a multidisciplinary group of implementation and improvement scientists, statisticians, health economists and behavioural and social science experts [13].

Development of the ImpRes tool involved an iterative process of:

#### 1a. Expert brainstorming and consensus-building sessions (July 2015–December 2015)

The CIS research team as well as international experts in the fields of implementation science and other healthcare disciplines, who were members of the CIS Scientific Advisory Panel (SAP), participated in three brainstorming sessions which informed the initial development and content of the ImpRes tool. Brainstorming sessions took place, and were the focus of, the CIS SAP face-to-face meetings over a 6-month period (July 2015–December 2015); additional input and feedback was sought from the CIS SAP virtually (via email). Content suggestions were collated by LH. Subsequently, the CIS research team participated in a consensus-building session which involved reviewing and considering the content suggestions for inclusion in the ImpRes tool.

The CIS SAP is an international multidisciplinary panel including clinicians and academics with expertise in implementation science, improvement science, social science, health policy, biostatistics, health economics, health service research and patient and public involvement. A full list of members of the CIS SAP is available in the ImpRes guide [14]. The CIS SAP convenes on a quarterly basis and offers critical appraisal and advice to the CIS research and strategy.

#### 1b. Identification of key methodological/conceptual literature containing guidance and recommendations for designing and evaluating implementation research (July 2015–March 2018)

Concurrently with the consensus-building brainstorming sessions, and informed by emerging ImpRes domains, a review of the literature was undertaken to identify key methodological/conceptual articles and reports containing guidance and recommendations relating to the design and evaluation of implementation research. Searching via PubMed and Google, articles and reports containing design guidance relating to the domains of ImpRes were identified and screened for relevance. Google was searched to identify gray literature and relevant content that would not have otherwise been identified through PubMed. For example, in addition to peer-reviewed publications, searching via Google allowed for the identification of websites, reports, webinars and blogs providing implementation research design guidance. The search was conducted between July 2015 and March 2018, with no date restrictions. Basic search terms reflected the emerging core ImpRes domains (e.g. ‘implementation outcomes’ and ‘implementation strategies’) and were used to identify relevant papers in PubMed and Google. Further, key articles by prominent authors/research groups leading work on specific domains of implementation science were also searched, including guidelines and recommendations relating to implementation outcomes (Enola Proctor) and implementation strategies (Byron Powell). Furthermore, identification of key literature was informed by the CIS SAP and the CIS implementation research teams’ expert knowledge. In this way, the ImpRes tool represents a consolidation and unification of key implementation science constructs informed by experts and key implementation science literature.

#### 1c. Additional specialist input into specific ImpRes domains (January 2016–March 2016)

Once the core domains of the ImpRes tool were established (i.e. through consensus amongst the CIS research team and SAP), additional specialist expert input into the content of specific domains of the ImpRes tool was sought. Specifically, the lead researcher (LH) met with NIHR CLAHRC South London specialists in patient and public involvement, and specialists in health economics (AH, a member of the research team), to review and co-design the content of the ‘patient and public involvement and engagement’ and ‘economic evaluation’ ImpRes domains, respectively. Specialist input was sought as PPI and economic evaluation are specialist fields, both of which are considered important facets of implementation research [15, 16].

#### 1d. Pilot testing and refinement (December 2015–May 2016)

The ImpRes tool was piloted by an experienced health services researcher without implementation science expertise (i.e. the intended target audience of the ImpRes tool). The ImpRes tool was completed independently by the researcher, using a research project that the researcher was leading on. Following completion, the lead researcher (LH) sought feedback on the ImpRes tool.

### Stage 2: application of the ImpRes tool (June 2016–August 2018)

The ImpRes tool was applied to 15 implementation and improvement science research projects prospectively (i.e. at project design stage) or retrospectively (i.e. after all project design decisions had been made and/or the project had been completed), across the NIHR CLAHRC South London and partner healthcare organizations. At the time of application, the ImpRes tool was completed by researchers at varying stages of developing implementation science expertise following the NIHR CLAHRC South London’s launch in 2014. The research projects focused on a wide range of healthcare areas and ranged from hybrid type 1 effectiveness-implementation studies to *pure* implementation research (see ‘Results’ section, Table 3).

To suit the needs and desires of researchers, a pragmatic and flexible approach was used to apply the ImpRes tool. For example, some researchers felt confident applying the ImpRes tool without the guidance of an implementation scientist (LH), whereas others lacked confidence in applying the tool independently and welcomed expert guidance. To suit the needs of all, researchers were given the option to either (1) complete the tool independently and then participate in a one-to-one feedback session after the implementation scientist had reviewed the completed ImpRes tool, or (2) complete the tool with direct facilitation provided by the implementation scientist. Facilitation consisted of explaining the rationale for developing the ImpRes tool, its aims and an overview of the ten domains (via one-to-one or group presentation sessions). Researchers applying the ImpRes tool were asked to complete ten sections corresponding to the ten domains of ImpRes. A number of domains require researchers to provide written responses in the form of a paragraph (e.g. in the ‘Implementation Strategies’ domain, researchers were asked to describe the implementation strategies that they intended to use/had used). Other sections involve completion of a checklist (e.g. ‘Implementation Outcomes’ and ‘Patient and Public Involvement and Engagement’ domains are formatted in the form of a checklist). Researchers were asked to complete ImpRes as best as they were able to. Although researchers were not asked to document the amount of time taken to complete the ImpRes tool, we are aware that many researchers invested a considerable amount of time completing ImpRes, especially when applied prospectively (i.e. at project design stage).

### Stage 3: Evaluation of the ImpRes tool (June 2016–August 2018)

#### 3a: Questionnaire (June 2016-August 2018)

After completing the ImpRes tool, participating researchers completed a short questionnaire indicating their level of agreement with 11 statements relating to the structure, content and the usefulness of the ImpRes tool. Responses were provided on 5-point Likert scales, ranging from 1 (*strongly disagree*) to 5 (*strongly agree*). For example, participants responded to the following statements relating to the structure and usefulness of ImpRes tool respectively: ‘The ImpRes tool is easy to understand’ and ‘The ImpRes tool is useful for identifying project areas where implementation science is lacking.’ Participants were also encouraged to provide free-text comments and critique. One questionnaire per ImpRes tool application was completed.

#### 3b: Download figures (April 2018-September 2018)

The ImpRes tool and supplementary guide were made freely available on the *King’s Improvement Science* (KIS) website [14] in April 2018 and the *Implementation Science Exchange* website [17] in May 2018. Since then, monthly download figures have been collected for both the ImpRes tool and guide on the KIS website (download figures from the *Implementation Science Exchange* website are not available to report as they are not currently collected by the website owners).

### Stage 4: development of the ImpRes supplementary guide (January 2017–April 2018)

During application of the ImpRes tool and informal feedback provided by researchers who had applied the tool, it became apparent that in order to maximize the potential benefits, usability and scalability of the ImpRes tool, a detailed guide to supplement its use was required. The guide provides the rationale for the inclusion of the ImpRes tool domains, guidance regarding the application of the ImpRes tool and directs researchers to further literature and specialist resources.

## Results

The process to develop and evaluate the ImpRes tool and supplementary guide is illustrated in Fig. 1. In what follows, we present the results corresponding to each stage of development and evaluation as described in the methods section and as depicted in Fig. 1. Results reporting the development of the ImpRes tool (stage 1) and guide (stage 4) correspond to the study aims of (1) identifying the core principles and concepts that research teams should consider when designing high-quality implementation research and (2) identifying and synthesizing key methodological/conceptual literature containing guidance and recommendations for designing and evaluating implementation research. Results reporting the application (stage 2) and evaluation of the ImpRes tool (stage 3) correspond to the aim of applying and evaluating a tool that guides researchers through the key principles and research design considerations of implementation science when designing studies.

### Overview of the ImpRes tool

**Fig. 2:**
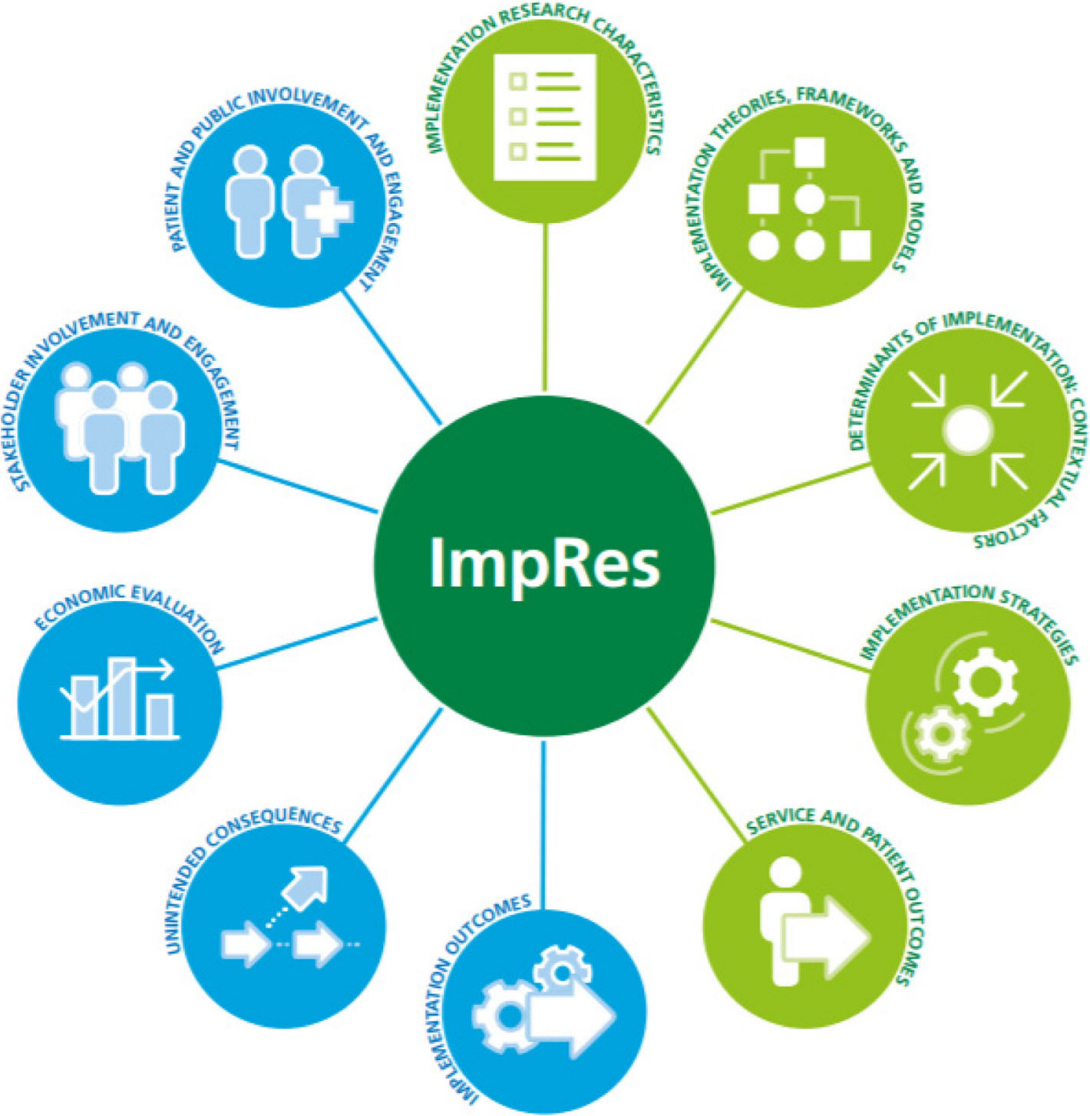
Domains of the ImpRes tool

The ImpRes tool contains ten domains that experts agreed, based on current evidence, cover the core principles and methods of implementation science that researchers should consider when designing implementation research (see Fig. 2).

### Stage 1: development of the ImpRes tool

#### 1a: Expert consensus-building brainstorming sessions

The ImpRes tool is organized into ten domains; each domain and the rationale underpinning its inclusion is presented in Table 1.

**Table 1:** ImpRes domains and rationale underpinning inclusion

ImpRes domain	Rationale underpinning inclusion
Domain 1: Implementation research characteristics	It has been argued that the research-to-practice gap is inflated by the tendency to conduct research in a step-wise manner from clinical efficacy research, followed by clinical effectiveness research, and lastly implementation research [18–21]. The benefits of simultaneously researching effectiveness and implementation to promote the more rapid and successful translation of clinical interventions have been previously described and a *hybrid effectiveness*-*implementation typology* proposed [22]. As such, the ImpRes tool encourages researchers to differentiate between studying the effectiveness of an evidence-based intervention (e.g. to improve a specific service or patient outcome) and studying the implementation of an intervention in a real-world setting (e.g. to identify barriers and facilitators to implementation and evaluate implementation success and strategies). The ImpRes tool urges researchers to categorize the degree of focus placed on evaluating the effectiveness of the intervention and/or implementation success (e.g. from *effectiveness*-*implementation hybrid* studies to *pure implementation* research [22]). The ImpRes tool and guide are not intended to be overly prescriptive in terms of implementation study design; different designs lend themselves to different project aims and objectives, as such there is not a one size fits all.
Domain 2: Implementation theories, frameworks and models	The use of implementation theories, frameworks and models is important in implementation research for multiple reasons including guiding implementation efforts, improving understanding of implementation determinants and providing a structure to synthesize findings [9]. Furthermore, the use of theories and frameworks enhances the generalizability of implementation efforts and help to build a cumulative understanding of the nature of change [23] enabling greater replication of evidence-based interventions in other settings. The underuse, superficial use and misuse of theories and frameworks have been described as posing a substantial scientific challenge for implementation science [9]. ImpRes does not endorse the use of any particular theory, framework or model as the aims and objectives of any given implementation research project will determine the appropriateness and relevance of particular implementation theories, frameworks and models. Depending on the aims and objectives of an implementation research project, it may be appropriate to apply more than one theory, framework or model. For example, if a project aims to identify and understand barriers and enablers to individual behavior change and simultaneously evaluate implementation efforts, the application of a determinant framework which focuses on individual level determinants, such as the theoretical domains framework (TDF) [24], as well as a framework, such as RE-AIM [25], that can be used to evaluate implementation efforts, would be appropriate. As such, the ImpRes tool encourages researchers to consider and apply implementation theories, frameworks or models in implementation research that are appropriate to the aims and objectives of the research.
Domain 3: Determinants of implementation	Implementation success is, in part, a function of the context in which implementation efforts occur. Without a clear understanding of the contextual factors likely to impede or facilitate implementation efforts, implementation strategies to overcome barriers and maximize facilitators will not be optimized, thereby reducing the likelihood of implementation success. The ImpRes tool specifically highlights the consolidated framework for implementation research (CFIR) [26], because CFIR brings together key constructs, derived from multiple implementation theories, that influence implementation efforts and is one of the most highly cited implementation determinant frameworks in the field [27]. Whilst the ImpRes tool highlights CFIR, it is important to note that ImpRes does not endorse the use of a particular determinant framework. Rather than simply describing the context in which implementation efforts occur, the ImpRes tool prompts research teams to design research to prospectively and systematically explore the factors that are likely to hinder or facilitate implementation efforts—including identification of factors commonly present across contexts that impede or facilitate health intervention implementation. Recent efforts to improve our understanding of implementation in given contexts have provided a more comprehensive, unified and structured conceptualization of context, extending beyond simply barriers and facilitators to implementation [28–30].
Domain 4: Implementation strategies	Described as constituting the ‘active ingredient’ and ‘how to’ components of implementation efforts, implementation strategies are regarded as having *unparalleled importance* in implementation science [31]. A program of research is currently underway to improve guidance regarding how best to select implementation strategies [8, 32, 33]. The ImpRes tool and guide aim to advise research teams to prospectively appraise the context in which they intend to implement evidence-based interventions and subsequently select contextually appropriate implementation strategies to maximize implementation success. The ImpRes tool and guide thus aim to facilitate explicit mapping of implementation strategies to prospectively identified barriers and facilitators to implementation efforts by signposting researchers to resources and tools designed to help researchers achieve this.
Domain 5: Service and patient outcomes	Researchers working in the field of applied health research will be familiar with, and understand the importance of, identifying, assessing and measuring relevant service and patient outcomes (commonly referred to as ‘client outcomes’ in the USA) to determine the efficacy and effectiveness of interventions. Unless conducting ‘pure’ implementation research [22], implementation outcomes [34], will be evaluated in addition to service and patient outcomes. The ImpRes tool and guide aim to highlight the limitations of only assessing service and patient outcomes when trying to understand and evaluate implementation. By making explicit the different types of outcomes an implementation study can assess, the ImpRes tool and guide facilitate the thinking that patient and service level outcomes are conceptually distinct from implementation outcomes—thus the need to assess the latter becomes prominent. In doing so, the ImpRes tool and guide aim to increase awareness that whilst the assessment and measurement of service and patient outcomes are essential to determine the clinical efficacy and effectiveness of an intervention, they are not sufficient for understanding implementation success, or failure of an intervention. For example, an intervention designed to reduce Emergency Department admissions might be found to be *ineffective* according to admission data (i.e. service outcomes). However, the intervention might subsequently be found to be effective, for example, when implemented as intended (i.e. implemented with fidelity).
Domain 6: Implementation outcomes	Implementation outcomes have been defined as ‘the effects of deliberate and purposive actions to implement new treatments, practices and services and are distinct from service and client (patient) outcomes.’ [34]. They may focus on a specific level of implementation, such as system, organization, innovation, provider and patient. Proctor et al. proposed a working taxonomy of eight implementation outcomes that may be assessed across all levels of a healthcare system, these include acceptability, appropriateness, feasibility, adoption, fidelity, penetration, implementation cost and sustainability [34]. Implementation outcomes may be assessed using validated measurement instruments, routinely collected data and qualitative methods. They should be assessed at salient stages of implementation (e.g. early for adoption, ongoing for penetration, late for sustainability). Furthermore, implementation strategies should be appropriately aligned to the implementation outcome(s) they intended to target and improve.
Domain 7: Economic evaluation	Implementation actions and strategies will inevitably consume scarce resources that could be put to other valued uses. Decision makers are therefore likely to want to gain insight into which options offer the greatest returns, in terms of patient or population health-related benefits, per $ of resource invested [35]. Economic evaluation provides a methodological framework for examining this question, and for adding further insight into the inter-dependencies between activities, cost, implementation outcomes and patient outcomes [36]. While the concept and importance of costing and consideration to cost-effectiveness in relation to implementation strategies is not particularly novel, to the best of our knowledge, the ImpRes tool and guide is the only implementation research tool of its type to provide guidance on data requirements for costing implementation strategies and how economic evaluation more generally should fit into a broader implementation evaluation plan.
Domain 8: Stakeholder involvement and engagement	The ImpRes tool explicitly urges researchers to view implementation research as a genuinely collaborative undertaking between researchers and stakeholders and therefore stresses the importance of involving and engaging stakeholders in the process of designing implementation research. Stakeholders are individuals and groups who are likely to be impacted or affected by implementation efforts; they may also be influential in determining the success or failure of implementation efforts (e.g. health service leaders or managers or commissioners). Involving and engaging stakeholders as part of the study design team helps to ensure that, for example, suitable implementation strategies and outcomes (both patient, service and implementation outcomes) are measured; and that an appropriate communication and dissemination plan are developed.
Domain 9: Patient and public involvement and engagement	Patient and public involvement (PPI) is widely considered a marker of high-quality research [37]. Evidence suggests that PPI in implementation research is lagging and has not matured to the same extent as PPI in other areas of health research and as such remains a largely untapped resource [38, 39]. The ImpRes tool prompts research teams to distinguish and consider PPI in isolation to other stakeholder involvement because of its critical importance in facilitating the implementation of research evidence into clinical practice and thus maximizing patient benefit and reducing health inequalities. As such, research teams are encouraged to view implementation research as a partnership between researchers and patients and thus involve patients and the public in designing implementation research.
Domain 10: Unintended consequences	Implementation efforts are often complex, challenging, and unpredictable. Unintended consequences (both positive and negative) are likely to occur as a result of implementation efforts; however, unintended consequences of implementation efforts are often not studied and/or under-reported. Research teams often focus their efforts on quantifying the intended and anticipated benefits of implementation efforts and fail to consider unintended and unanticipated consequences. The ImpRes tool prompts research teams to be mindful of and explore the potential unintended consequences of implementation efforts.

#### 1b: Identification of key methodological/conceptual literature containing implementation research design guidance and recommendations

Concurrently with the consensus-building brainstorming sessions and informed by emerging ImpRes domains (presented above), key methodological and conceptual articles and reports (both peer-reviewed and non-peer-reviewed) were identified in relation to each of the 10 ImpRes domains. These are presented in Table 2.

**Table 2:** Key methodological/conceptual articles, reports and resources identified that influenced the content of the ImpRes tool and/or guide

ImpRes domain	Key methodological/conceptual articles, reports and resources (peer-reviewed and non-peer reviewed) providing research design guidance and recommendations
Domain 1: Implementation research characteristics	Useful references: peer-reviewed publications
	• Brown et al. An Overview of Research and Evaluation Designs for Dissemination and Implementation. Annu Rev. Public Health. 2017;38:1–22 [40].
	• Curran et al. Effectiveness-implementation hybrid designs: combining elements of clinical effectiveness and implementation research to enhance public health impact. Med Care. 2012;50(3):217–26 [22].
	Useful resources: resources including blogs, webinars and websites
	• National Implementation Research Network. Stages of Implementation. http://nirn.fpg.unc.edu/learn-implementation/implementation-stages [41].
Domain 2: Implementation theories, frameworks and models	Useful references: peer-reviewed publications
	• Birken et al. Criteria for selecting implementation science theories and frameworks: results from an international survey. Implement Sci. 2017;12(1):124 [9].
	• Eccles et al. Changing the behavior of healthcare professionals: the use of theory in promoting the uptake of research findings. J Clin Epidemiol. 2005;58(2):107–12 [42].
	• Nilsen. Making sense of implementation theories, models and frameworks. Implement Sci. 2015;10:53 [43].
	• Tabak et al. Bridging research and practice: models for dissemination and implementation research. Am J Prev Med. 2012;43(3):337–50 [44].
	Useful resources: resources including blogs, webinars and websites
	• Implementation Science Exchange. https://impsci.tracs.unc.edu [17].
	• The Consolidated Framework for Implementation Research (CFIR) website. www.cfirguide.org [45].
	• Reach Effectiveness Adoption Implementation Maintenance (RE-AIM) framework website. http://re-aim.org [46]. • Normalisation Process Theory. http://www.normalizationprocess.org/. Accessed 12 May 2019. [47].
Domain 3: Determinants of implementation	Useful references: peer-reviewed publications
	• Chaudoir et al. Measuring factors affecting implementation of health innovations: a systematic review of structural, organizational, provider, patient, and innovation level measures. Implement Sci. 2013;8:22 [48].
	• Craig et al. Developing and evaluating complex interventions: the new Medical Research Council guidance. BMJ. 2008;337:a1655 [49].
	• Damschroder et al. Fostering implementation of health services research findings into practice: a consolidated framework for advancing implementation science. Implement Sci. 2009;4:50 [26].
	• Flottorp et al. A checklist for identifying determinants of practice: A systematic review and synthesis of frameworks and taxonomies of factors that prevent or enable improvements in healthcare professional practice. Implement Sci. 2013;23;8:35 [50].
	• Nilsen. Making sense of implementation theories, models and frameworks. Implement Sci. 2015;10:53 [43].
	Useful resources: resources including blogs, webinars and websites
	• Health Foundation webinar: Quality Improvement and the role of context and how to manage it. www.health.org.uk/webinar-quality-improvement-role-context-and-how-manage-it [51].
	• Health Foundation. Perspectives on Context: A selection of essays considering the role of context in successful quality improvement. https://www.health.org.uk/sites/health/files/PerspectivesOnContext_fullversion.pdf [52].
	• The Consolidated Framework for Implementation Research (CFIR) website. www.cfirguide.org [45].
Domain 4: Implementation strategies	Useful references: peer-reviewed publications
	• Powell et al. Methods to improve the selection and tailoring of implementation strategies. J Behav Health Serv Res. 2017;44(2):177–194 [53].
	• Proctor et al. Implementation strategies: recommendations for specifying and reporting. Implement Sci. 2013;8:139 [31].
	• Waltz et al. Use of concept mapping to characterize relationships amongst implementation strategies and assess their feasibility and importance: results from the Expert Recommendations for Implementing Change (ERIC) study. Implement Sci. 2015;10:109 [33].
Domain 5: Service and patient outcomes	Useful references: peer-reviewed publications
	• Proctor et al. Outcomes for implementation research: conceptual distinctions, measurement challenges, and research agenda. Adm Policy Ment Health. 2011;38(2):65–76. [34]
Domain 6: Implementation outcomes	Useful references: peer-reviewed publications
	• Clinton-McHarg et al. Psychometric properties of implementation measures for public health and community settings and mapping of constructs against the Consolidated Framework for Implementation Research: a systematic review. Implement Sci. 2016;11:148 [54].
	• Lewis et al. Outcomes for implementation science: an enhanced systematic review of instruments using evidence-based rating criteria. Implement Sci. 2015;10:155 [55].
	• Proctor et al. Outcomes for implementation research: conceptual distinctions, measurement challenges, and research agenda. Adm Policy Ment Health. 2011;38(2):65–76 [34].
	Useful resources: resources including blogs, webinars and websites
	• The Society for Implementation Research Collaboration (SIRC) Implementation Outcomes Repository website. https://societyforimplementationresearchcollaboration.org/ [56].
	• Grid-Enable Measures (GEM) database website. https://www.gem-beta.org/Public/Home.aspx [57].
	• National Institute for Health National Cancer Institute. Advanced Topics for Implementation Science Research: Measure Development and Evaluation webinar. https://www.youtube.com/watch?v=dGXVhRQXiz4 [58].
Domain 7: Economic evaluation	Useful references: peer-reviewed publications
	• Thompson et al. The cost-effectiveness of quality improvement projects: a conceptual framework, checklist and online tool for considering the costs and consequences of implementation-based quality improvement. J Eval Clin Pract. 2016;22(1):26–30 [35].
	• Mason et al. When is it cost-effective to change the behavior of health professionals? JAMA. 2001;286(23):2988–92 [59].
	Useful resources: resources including blogs, webinars and websites
	• Blog: Theory and practice: Finding common ground between health economics and implementation science. https://blogs.biomedcentral.com/on-health/2014/12/18/theory-and-practice-finding-common-ground-between-health-economics-and-implementation-science/[60].
	• Checklist and Online Resource (PCEERT) for Considering the Value of Implementation-Based Quality Improvement [35].
Domain 8: Stakeholder involvement and engagement	Useful references: peer-reviewed publications
	• Rycroft-Malone et al. Collaborative action around implementation in collaborations for leadership in applied health research and care: Towards a programme theory. J Health Serv Res Policy. 2013 18(3 Suppl):13–26 [38].
	Useful resources: resources including blogs, webinars and websites
	• Blog: Where are the stakeholders in implementation science? http://nirn.fpg.unc.edu/where-are-stakeholders-implementation-science [61].
Domain 9: Patient and public involvement and engagement	Useful references: peer-reviewed publications
	• Burton et al. An Untapped Resource: Patient and Public Involvement in Implementation Comment on “Knowledge Mobilization in Healthcare Organizations: A View from the Resource-Based View of the Firm”. Int J Health Policy Manag. 2015;4(12):845–7 [39].
	• Callard et al. Close to the bench as well as at the bedside: involving service users in all phases of translational research. Health Expect. 2012;15(4):389–400 [62].
	• Ocloo J et al. From tokenism to empowerment: progressing patient and public involvement in healthcare improvement. BMJ Qual Saf. 2016;25(8):626–32 [63].
	Useful resources: resources including blogs, webinars and websites
	• National Institute for Health (NIHR) Report: Going the extra mile: improving the nation’s health wellbeing through public involvement in research. https://www.nihr.ac.uk/patients-and-public/documents/Going-the-Extra-Mile.pdf [64].
	• National Institute for Health (NIHR) INVOLVE website. http://www.invo.org.uk/ [65].
	• Hayes H et al. National Institute for Health (NIHR) INVOLVE Briefing notes for researchers: public involvement in NHS, public health and social care research. http://www.invo.org.uk/resource-centre/resource-for-researchers/ [66].
	• National Institute for Health (NIHR) INVOLVE Jargon Buster. http://www.invo.org.uk/resource-centre/jargon-buster/ [67].
Domain 10: Unintended consequences	Useful references: peer-reviewed publications
	• Merton R. The unanticipated consequences of purposive social action. Am Sociol Rev. 1936;1:894e904 [68].
	Useful resources: resources including blogs, webinars and websites
	• The Office of the National Coordinator for Health Information Technology (ONC). Online module: Introduction to Unintended Consequences. https://www.healthit.gov/unintended-consequences/content/module-i-introduction-unintended-consequences.html [69].

#### 1c: Additional specialist input into specific ImpRes domains

After consulting with experts in the field of PPI and health economics, several refinements to the ImpRes tool were made. For example, after consulting with an expert in PPI in health services research, to ensure that researchers considered and described involvement and engagement opportunities at different stages of the research cycle (i.e. from *identifying and prioritizing research topics* to *evaluating impact*), and to distinguish between the different levels of involvement (i.e. *consultation* to *collaboration*), responses were tabulated rather than requesting researchers to describe planned involvement and engagement activities. Furthermore, four additional questions were included in the ‘Patient and Public Involvement’ section of the ImpRes tool, including, but not limited to, whether the patients/service users that researchers intended to involve in their research have formal research training.

#### 1d: Pilot testing and refinement

The tool did not undergo any significant refinements after piloting. In addition to the refinements based on specialist expert input (described above), additional feedback received by the CIS SAP at subsequent meetings (March 2016 onwards) and discussions amongst the CIS Research team, a number of refinements were made to the ImpRes tool. For example, to improve the usability of the tool, a number of sections were tabulated (e.g. implementation outcomes, service and patient outcomes, and economic evaluation domains).

### Stage 2: application of the ImpRes tool

The ImpRes tool was applied to 15 implementation and improvement science projects, either prospectively or retrospectively. The ImpRes tool was fully completed for 14 projects and partially completed for one project. The ImpRes tool was applied independently by researchers (i.e. without expert guidance) to 14 projects. ImpRes was applied with the guidance of an implementation scientist (LH) to the remaining project. The tool developers are part of the research organization in which the tool was applied and evaluated. As a large research organization, the relationship between the tool developers and the researchers that applied and evaluated the ImpRes tool was variable. In the majority of cases, the application of the ImpRes tool was the first meeting between the tool developers and the lead researcher. The research projects covered a diverse range of clinical areas, including diabetes, mental health, dementia and maternity and women’s health. Two hybrid type 1, five hybrid type 2, three hybrid type 3 and five implementation research projects, as defined by Curran et al., [22], were included. Additional details of each project can be found in Table 3.

**Table 3:** Application of the ImpRes tool

Project title and *CLAHRC South London Research theme* Project web page (if available)	Healthcare area	Research stage at which the ImpRes tool was applied (prospective or retrospective)	Study field and design of study^a^	Position of researcher(s) completing the ImpRes tool and ImpRes application details
Prevention of Preterm Birth Plus Improving Women’s Experiences; *Maternity and Women*’*s Health research theme* https://www.medscinet.net/POPPIE	Maternity and women’s health	Prospective application	Implementation Science project; *Hybrid type 2*	Midwife researchers Completed independently followed by face-to-face feedback session with three members of the research team.
Developing, implementing and evaluating tobacco dependence treatment for people with psychosis and staff training pathways; *Psychosis research theme* No website available	Mental health	Retrospective application	Implementation Science project: *Hybrid type 3*	Senior post-doctoral researcher Completed independently. Feedback session offered but not accepted.
Service Evaluation of Alcohol Identification and Brief Advice Direct to the Public; *Alcohol theme*, *Health Innovation Network South London*^b^ Project website: https://healthinnovationnetwork.com/projects/iba-direct-service-evaluation/	Alcohol	Retrospective application	Improvement/spread project: *Hybrid type 3*	Senior Project Manager Completed independently, feedback session completed.
Evaluation of the delivery of a clinical trial intervention to improve hypoglycemia control in type 1 diabetes: Applying an implementation science approach; *Diabetes Research theme* Project website: http://www.clahrc-southlondon.nihr.ac.uk/centre-implementation-science/our-research/evaluating-harpdoc-trial-aimed-helping-people-type-1-diab	Diabetes	Prospective application	Implementation Science project: *Hybrid type 1*	Post-doctoral researcher Completed independently and feedback session completed.
DeAR-GP dementia assessment referral to GP; *Dementia theme, Health Innovation Network South London*^b^ Project website: https://healthinnovationnetwork.com/projects/dear-gp/	Dementia	Prospective application	Improvement/spread project: *Implementation Research*	Senior Project Manager Completed independently and feedback session completed.
Development of an atrial fibrillation/atrial flutter care pathway in the cardiac device clinic; *King*’*s Improvement Science*, *Centre for Implementation Science*. *Project website*: *http://www.kingsimprovementscience.org/kis*-*project*-*3*-*end*-*of*-*project*-*november*-*2016*	Cardiac services	Retrospective application	Improvement Science project; *Hybrid type 3*	Research worker Completed independently and feedback session completed.
ICARUS Quality improvement project—implementing a safety briefing in critical care; *King*’*s Improvement Science*, *Centre for Implementation Science.* Project website: http://www.kingsimprovementscience.org/kis-project-5/end-of-project-november-2016	Critical care	Retrospective application	Improvement Science project: *Implementation Research*	Health Service Researcher/Improvement Science specialist Completed independently and feedback session completed.
iMobile—improving critical care discharge summaries; *King*’*s Improvement Science, Centre for Implementation Science.* Project website: http://www.kingsimprovementscience.org/kis-project-1-end-of-project-update-february-2015	Critical care	Retrospective application	Improvement Science project; *Implementation Research*	Health Service Researcher/Improvement Science Specialist Completed independently. Feedback session not completed.
Improving Lung Health in Addictions Services; *King*’*s Improvement Science, part of the Centre for Implementation Science*. Project website: http://www.kingsimprovementscience.org/kis-project-4-end-of-project-november-2016	Drug and Alcohol Services	Retrospective application	Improvement Science project; *Implementation Research*	Research worker Completed independently and feedback session completed.
Implementing optimal falls risk assessment in memory services; *Dementia theme*, *Health Innovation Network South London*^b^ Project website: https://healthinnovationnetwork.com/projects/implementing-optimal-falls-risk-assessment-in-memory-services/	Dementia	Prospective application	Improvement/spread project: *Hybrid type 2*	Innovation Fellow Completed independently and feedback session completed.
Technology Enabled Patient Consultation; *Technology and Information theme*; *Health Innovation Network South London*^b^ Project website: *No project website available*	Technology and Information	Prospective application	Improvement/spread project; *Hybrid type 2*	Technology Project Manager Completed independently and feedback session completed.
Reducing Catheter Associated Urinary Tract Infections (CAUTIs) in South London to improve patient safety, experience and value; *Patient Safety theme, Health Innovation Network South London*^b^ Project website: https://healthinnovationnetwork.com/projects/catheter-care/	Patient Safety	Prospective application	Improvement/spread project; *Hybrid type 2*	Clinical Nurse Specialist Completed independently and feedback session completed.
A Process Evaluation of the Implementation and Effectiveness of an Intensive Care Unit Telemedicine Programme; *King*’*s Improvement Science, Centre for Implementation Science.* *No project website available*	Intensive care	Prospective application	Improvement Science project; *Hybrid type 2*	Post-doctoral research fellow Completed independently and feedback session completed.
Process evaluation of a violence reduction programme in inpatient psychiatric wards. *King*’*s Improvement Science*, *Centre for Implementation Science.* Project website: http://www.kingsimprovementscience.org/dina-poursanidou	Mental health	Retrospective application	Improvement Science project; *Implementation Research*	King’s Improvement Science Fellow Completed with lead researcher
Walk this Way: a coaching intervention to reduce sedentary behaviour in people with severe mental illness; Psychosis research theme Project website: http://www.clahrc-southlondon.nihr.ac.uk/psychosis/physical-health	Mental health	Prospective application	Implementation Science project; *Hybrid Type 1*	Post-doctoral researcher Completed independently and feedback session completed.

^a^Design of study: clinical efficacy research; clinical effectiveness research; hybrid type 1: hybrid type 2; hybrid type 3; implementation research as defined by Curran et al., 2012 [22]

^b^Partner organization of NIHR CLAHRC South London

^c^ImpRes tool partially completed

### Stage 3: evaluation of the ImpRes tool

#### 3a. Questionnaire

Thirteen out of 15 questionnaires were completed. Evaluative feedback from researchers on the structure, content and usefulness of the ImpRes tool is reported in Table 4. In all but one case (where the researcher stated that ‘*The ImpRes tool is too long’*), the median rating for statements relating to the structure and content and the usefulness of the ImpRes tool were above the scale mid-point (3) thereby indicating that researchers who applied the ImpRes tool viewed the structure, content and usefulness of the tool favorably.

**Table 4:** Evaluation of the ImpRes tool

Statements	Median (IQR)
Structure and content	
The ImpRes tool is easy to understand	4 (3–4)
The ImpRes tool is time consuming to complete	4 (3–4)
The ImpRes tool is too long	3 (2–4)
The order of the questions is logical	4 (4–4)
The ImpRes tool covers the key components that should be considered when designing/conducting an implementation project	4 (4–4)
Usefulness	
The ImpRes tool is a useful for self/project team reflection regarding implementation research	5 (4–5)
The ImpRes tool is useful for identifying project areas where implementation science is lacking	5 (4–5)
ImpRes is a useful tool to strengthen implementation science in projects	5 (4–5)
Giving project teams feedback on their project based on the ImpRes tool would be useful for improving the quality of implementation research	4 (4–5)
ImpRes is a useful tool to audit the quality of implementation research	4 (4–4)
ImpRes is a useful tool in identifying projects that should be adopted by CLAHRC South London^a^, *N* = 12^b^	4 (3–5)

1 = strongly disagree, 2 = disagree, 3 = neither agree nor disagree, 4 = agree, 5 = strongly agree

^a^Research organization in which ImpRes was developed, applied and evaluated

^b^*N* = 13 unless otherwise stated

### Free text comments provided by researchers after completing the ImpRes tool

Five out of 13 researchers who applied the ImpRes tool, to 15 individual projects, provided free-text comments. Suggestions for improvement included expanding the glossary at the end of the ImpRes tool: ‘Helpful to provide definitions of “implementation/project team” and “stakeholders” distinction not clear. Also, helpful to provide definitions of ‘research project’ and ‘improvement project’ (Researcher 3)’. One researcher suggested that the layout of the ImpRes tool needed modification; ‘I think the layout will need some tweaking to be a bit more user friendly. I felt that I needed help to clarify where which responses should go where’ (Researcher 8). Whilst some researchers thought that the ImpRes tool was too long, one researcher commented that ImpRes was necessarily long: ‘The length [of the ImpRes tool] and level of detail are necessary to capture the complexity of the issues explored’ (Researcher 15). Furthermore, acknowledging that the ability to successfully conduct and complete an implementation project depends on the skills, expertise and experiences of the research team, one researcher suggested including an additional question: ‘There is maybe a question missing about the skill set of the project teams’ (Researcher 8).

#### 3b: Download figures

**Fig. 3:**
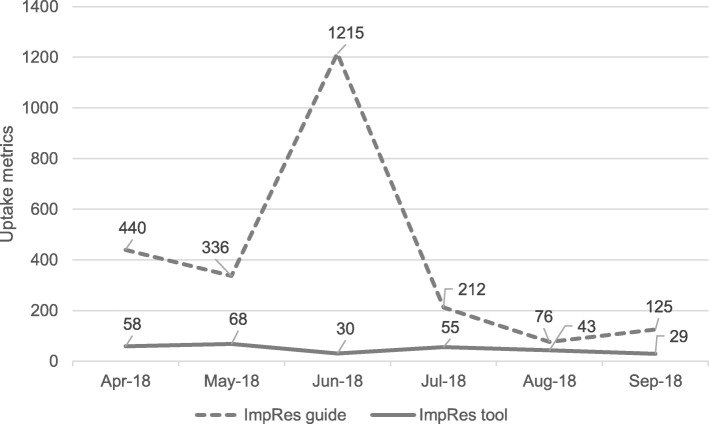
Download figures for the ImpRes tool and guide

Figure 3 displays the download figures for the ImpRes tool and guide over a 6-month period (April–September 2018). In total, the ImpRes tool and guide were downloaded 2687 times. The ImpRes guide was downloaded 1215 times in June 2018, representing a substantial increase in comparison to other months. We are aware that the ImpRes tool and guide were presented as part of a workshop at a large international conference and received social media attention (twitter).

### Stage 4: Development of the ImpRes supplementary guide

The evaluation data collected on the ImpRes tool (described in stage 3: evaluation of the ImpRes tool) led to the development, and informed the content, of the ImpRes supplementary guide. The guide is intended to be used in conjunction with the ImpRes tool. The guide aims to facilitate the use of the ImpRes tool, highlight the importance of implementation science in optimizing the successful implementation of evidence-based interventions into clinical practice, define terminology commonly used in the implementation science literature and direct researchers to relevant literature and online resources that can be used to design implementation research. Throughout the guide, several features designed to support researchers are presented. These include a ‘jargon buster’ and ‘useful resources’ feature. Each feature, rationale for inclusion and feature examples are presented in Table 5. Together with the ImpRes tool, the ImpRes supplementary guide is free to download via the King’s Improvement Science website [14] and the Implementation Science Exchange website [17].

**Table 5:** ImpRes guide features, rationale for inclusion and feature examples

ImpRes guide features	Rationale for inclusion	Feature examples
Jargon buster	Implementation science has been described as a ‘tower of babel’ [70]. To address this issue, a ‘jargon buster’, providing widely accepted definitions of commonly used terms in implementation science is included in the ImpRes guide. It is hoped that this feature will further promote attempts, and the need, to bring consistency to terminology used in the field.	ImpRes domain: Implementation outcomes
		Implementation outcomes have been defined as ‘the effects of deliberate and purposive actions to implement new treatments, practices, and services and are distinct from service and client (patient) outcomes’ Proctor et al., 2011 [34].
		ImpRes component: Implementation Strategies
		Implementation strategies are ‘methods or techniques used to enhance the adoption, implementation, and sustainability of a clinical programme, practice or intervention’ Proctor et al., 2013 [31].
Top tips	Implementation science has been described as a growing but not well understood field of health research [71]. To help address this challenge, a ‘top tips’ feature exists to help researchers design high-quality implementation research. Whilst these ‘tips’ are embedded within the literature, it is hoped that the ‘top tips’ feature will ensure that existing design recommendations will be brought to the forefront of researchers’ minds when designing implementation research.	ImpRes domain: Implementation theories, framework and models
		Depending on the nature and aim(s) of your implementation research, it might be appropriate to use more than one theory, framework or model. For example, if your research aims to identify barriers to implementation as well as evaluating implementation, selecting an implementation determinant framework as well as an implementation evaluation framework would be appropriate.
Keep an eye out	Implementation science is a rapidly advancing field with much work currently being conducted to address lack of design guidance. The ‘keep an eye out’ feature signposts researchers’ to research that is currently being conducted that is not yet completed/published.	ImpRes domain: Implementation strategies
		A team of implementation scientists are currently developing the Implementation Technique Selection tool to ‘guide the selection of individual strategies that can be bundled or packaged together into an overall implementation strategy’. For more information, click here: http://cfirguide.org/techniques.html [72]
Useful references	Many peer-review publications exist that have the potential to increase knowledge and understanding and guide methodological decisions in implementation research. It is hoped this feature will signpost researchers to key peer-reviewed publications that can be used to guide the design of implementation research.	ImpRes domain: Implementation theories, framework and models
		Tabak et al. Bridging research and practice: models for dissemination and implementation research. Am J Prev Med. 2012;43:337–350 [44].
		Nilsen P. Making sense of implementation theories, models and frameworks. Imp Sci. 2015;10:53 [43].
Useful resources	In addition to peer-reviewed publications, many online resources exist that have the potential to increase knowledge and understanding and facilitate the design of implementation research. The ‘useful resources’ feature signposts researchers to blogs, webinars and websites.	ImpRes domain: Implementation theories, frameworks and models
		Website: Consolidated Framework for Implementation Research (CFIR) website (http://www.cfirguide.org/) [45].
		ImpRes domain: Implementation outcomes
		Webinar: Advanced Topics for Implementation Science Research: Measure Development and Evaluation. (https://www.youtube.com/watch?v=dGXVhRQXiz4) [58].
Link between ImpRes domains	Although presented separately, the ten ImpRes domains should be viewed as interlinked and overlapping rather than discrete. Here, we make these interlinks explicit.	ImpRes domains: Service and Patient Outcomes
		Stakeholder Involvement and Engagement: Selecting service and/or patient outcomes to assess and measure should be informed by involving stakeholders. Measuring and assessing outcomes important to stakeholders can have a significant impact on adoption, implementation and sustainment of evidence-based practice.

## Discussion

We developed the ImpRes tool and supplementary guide to help researchers design high-quality implementation research. Development of the tool and guide was informed by a consensus-building brainstorming process, involving an international multidisciplinary expert panel, and identification of key methodological and conceptual literature containing design guidance and recommendations. The ImpRes tool contains ten core domains, representing core implementation science principles and concepts that should be reviewed and considered when designing implementation research. Whilst we recommend that all components of ImpRes are worthy of consideration by research teams when designing implementation research, we recognize that not all sections will be applicable to, or feasible to explore, in every implementation study. Rather, each ImpRes domain should be considered strategically, in the context of the research aims and objectives, to determine applicability, importance and feasibility. For example, *implementation research*, as defined by Curran et al., 2012, which is focused on the adoption or uptake of a clinical intervention and as intervention effectiveness is typically assumed, gathering information on the clinical intervention’s impact on relevant outcomes is typically not assessed, as such ImpRes’ ‘Service and Patient Outcomes’ domain would not be applicable to include in such research. To the best of our knowledge, the ImpRes tool and supplementary guide is the only currently available comprehensive research design instrument which synthesizes guidance and recommendations for designing high-quality implementation research.

We envisage that the ImpRes tool and supplementary guide will increase users’ confidence and ability to design high-quality implementation research. The results of the present study support this vision; researchers applying the ImpRes tool found the tool to be useful in identifying areas where implementation science was lacking in research projects and in identifying how the methodology of projects could be improved. We anticipate that research teams who use the ImpRes tool will be more likely to consider and integrate core principles and concepts of implementation science when designing and evaluating the implementation of evidence-based health interventions. We aspire that, in turn, this will contribute to more successful implementation and evaluation of evidence-based interventions.

### Strengths and limitations

Strengths of our research include the application of the ImpRes tool to a number of implementation and improvement science research projects, by researchers with varying levels of implementation and improvement science expertise and across a diverse range of health care areas, including physical and mental health. Our initial evaluation of the ImpRes tool found that the tool is acceptable and feasible to apply. Evaluation of the ImpRes tool by researchers who applied the tool viewed the structure, content and usefulness of the tool very favorably; all but one *structure* evaluation item (‘the ImpRes tool is too long’) had a median score ≥ 4/5 and all *usefulness* evaluation items had a median score ≥ 4/5. Our initial dissemination efforts (via the *King*’*s Improvement Science* and *Implementation Science Exchange* websites) [14, 17] suggest that the ImpRes tool and guide fill an important capacity building gap; over a 6-month period, the ImpRes tool and guide have been downloaded over 2600 times.

These findings, however, must be interpreted with some caution. ImpRes was evaluated by researchers with varying levels of implementation and improvement science expertise and it could be argued that those with limited implementation science expertise are not best placed to evaluate the content, structure and usefulness of the ImpRes tool. That said, the acceptability and adoption of the ImpRes tool and guide depends on the views of this large cohort of researchers, the primary intended end user, who have expertise in applied health research but lack specialist implementation science training. Although our evaluation data to date indicates that experienced applied health researchers, with varying levels of implementation expertise, believe that ImpRes is likely to lead to better designed implementation studies, more robust evidence is needed to assess whether the ImpRes tool and guide could result into better designed implementation research and improved effectiveness of interventions. Whilst we plan to investigate the impact of using the ImpRes tool and guide on the quality of implementation research in the future, here we report the preliminary findings and our reflections on the ImpRes tool and guide.

Furthermore, we identified a number of barriers that are likely to affect adoption, implementation and sustainment of the ImpRes tool, including a lack of awareness amongst applied health researchers of implementation science to improve the implementation of evidence-based interventions. Moreover, ImpRes was applied and evaluated within a research organization: NIHR CLAHRC South London. Future evaluation studies should explore the generalizability and scalability, as well as the barriers and enablers, of the ImpRes tool and guide in other research settings (e.g. outside of large, multi-million-pound funded research organizations such as NIHR CLAHRCs and research cutting across health and social care settings). The researchers who developed the ImpRes tool developed and distributed the questionnaire that was used for the preliminary evaluation and analyzed the questionnaire data returned. Although a point was made of welcoming participants’ comments and critiques to help us to modify/refine the content of ImpRes tool, we are aware a social desirability bias may be present in the evaluation data.

#### Reflections on implementing the ImpRes tool

##### Barriers to implementing the ImpRes tool

Applying the ImpRes tool retrospectively (i.e. to research projects that had already been completed or were underway) proved to be an unmotivating task to researchers and is likely to explain the reluctance and lack of engagement of a number of researchers/research themes that were approached, but declined, to apply the ImpRes tool to their projects. This is not unexpected considering that research projects already completed would not directly benefit from applying the ImpRes tool, i.e. it was not possible to amend and improve the design of implementation or improvement science research as the research was already underway or had been completed. Like many applied health researchers, several researchers lacked awareness/familiarity with implementation science. This is likely to have resulted in some reluctance to commit the time needed to complete the ImpRes tool. Whilst we believe that the content of the ImpRes tool and guide is appropriate across all types of implementation research, the presentation of information may need to be revised, perhaps shortened, to better suit practitioner-researchers based outside of an academic research organization. Without education to establish awareness of the importance of implementation science in implementing evidence-based interventions, this barrier is likely to prevent the adoption, implementation and sustainability of the ImpRes tool. Furthermore, the ImpRes tool was perceived by some as a research-heavy tool targeted more at large-scale academic research rather than more applied, small-scale, pragmatic projects (e.g. improvement/spread projects). Such perceptions were expressed predominately by those who applied the ImpRes tool to projects being conducted outside of a university setting (i.e. Health Innovation Network) rather than those who applied the tool to projects being conducted within a university setting (i.e. King’s College London). Again, this is likely to have resulted in some reluctance to commit the time needed to complete the ImpRes tool.

##### Facilitators to implementing the ImpRes tool

Prospective application to projects that were in the design phase, and amenable to design change, and to grant applications that were being drafted for submission, perhaps unsurprisingly proved to be a factor motivating the application of ImpRes. The benefits of applying ImpRes were clear and immediate. Researchers who were actively encouraged by principal investigators (PIs) to apply the ImpRes tool were far more likely and motivated to do so than researchers who were approached on an individual basis and who had not received direct encouragement from PIs. Furthermore, researchers who were aware and interested in implementation science and implementation research yet felt they lacked the knowledge and skills to design implementation research welcomed the ImpRes tool, together with the support and facilitation of the lead researcher (LH), to structure the process of designing implementation research.

### Planned ImpRes research and development

Literature in the field of implementation science is a rapidly advancing. To ensure the ImpRes tool and guide include the most up-to-date guidance for designing and evaluating implementation research, we plan to review and update the tool and guide annually; the next review will be in April 2019. We are currently designing a formal international expert Delphi study, drawing upon a wider international expert panel, to formally content validate and refine the ImpRes tool and supplementary guide. Alongside this, we are currently developing quantitative scoring criteria for each of the ImpRes domains. The scoring criteria will provide a systematic and transparent rating system that will allow us to empirically determine whether applying the ImpRes tool and guide improves the quality of implementation research. We hope the scoring criteria will also be of benefit to multiple implementation research stakeholders (e.g. researchers, funders and decision-makers). For example, we envisage this scoring system to be of use to funders and decision-makers wishing to evaluate the quality of implementation research proposals allowing the differentiation between lower and higher quality implementation research proposals. Additionally, we hope that the scoring criteria will be of use and support researchers to improve the quality of their implementation research proposal by identifying project areas that require improvement. Furthermore, researchers who have implementation science expertise may also benefit from using the ImpRes tool and guide. The initial intention of the ImpRes tool and guide at the inception stage was to support those researchers who have no or limited expertise in implementation science research; however, we acknowledge and plan to evaluate the benefits of applying the ImpRes tool and guide for applied health researchers who have implementation science expertise.

## Conclusion

We have developed a new and promising educational tool and supplementary guide to help overcome the many challenges that applied health researchers face when attempting to design implementation research. We believe that adopting the ImpRes tool and guide will improve the quality of implementation research, in turn advancing the field and leading to optimized implementation of evidence-based interventions and ultimately improved service and patient outcomes.

## Abbreviations

CIS SAP: Centre for Implementation Science Scientific Advisory Panel
CIS: Centre for Implementation Science
CLAHRC: Collaboration for Leadership in Applied Health Research and Care
ImpRes tool: Implementation Science Research Development tool
IQR: Interquartile range
NIHR: National Institute for Health Research
PPI: Patient and public involvement
